# Impact of Temperature Variation on the Biological Traits and Lifecycle of *Spodoptera exigua* (Lepidoptera: Noctuidae): A Meta-Analysis Approach

**DOI:** 10.3390/insects16020155

**Published:** 2025-02-03

**Authors:** Honghua Zhang, Danping Xu, Xingqi Deng, Zhiqian Liu, Zhipeng He, Junhao Wu, Zhihang Zhuo

**Affiliations:** College of Life Science, China West Normal University, Nanchong 637002, China; honghua_zhang@foxmail.com (H.Z.); xudanping@cwnu.edu.cn (D.X.); deng.xinqi@foxmail.com (X.D.); qnhtvxhp319123@foxmail.com (Z.L.); zhipeng_hh@foxmail.com (Z.H.); wujunhao0824@gmail.com (J.W.)

**Keywords:** *Spodoptera exigua*, climate impact, pest management strategies, invasive species, meta-analysis, survival analysis

## Abstract

*Spodoptera exigua* is a pest of considerable economic importance; however, detailed research into its ecological adaptability in newly invaded habitats is limited. This research performed a comprehensive analysis of the life history characteristics of *S. exigua* under varying temperature, photoperiod, and humidity conditions. A total of 264 studies that met the inclusion criteria were included in the analysis, and the data were examined using random-effects model, fixed-effects model, and meta-regression analysis techniques. The findings reveal that when temperatures exceed 20 °C, several biological parameters of *S. exigua* significantly increase, with the highest biological activity observed at 33 °C. As temperature rises, the duration of each developmental phase significantly decreases, accompanied by a reduction in the average number of eggs produced by females and a shorter pre-oviposition period. In addition, the pupal development period is shortened, resulting in a longer adult lifespan. By considering environmental variables such as temperature, photoperiod, and relative humidity, we identified the optimal conditions for the survival of each developmental stage of *S. exigua*. These results provide a foundation for predicting the population dynamics of this pest and contribute to the development of more effective pest control strategies.

## Simple Summary

*Spodoptera exigua* is a significant pest of crops, but its ability to adapt to new climates remains poorly understood. This study investigates how temperature, light cycle, and humidity affect the beet armyworm’s lifecycle. By analyzing 264 data points from 33 published studies, we found that warmer temperatures, particularly above 20 °C, significantly enhance the beet armyworm’s physiological functions. As temperatures increase, the developmental stages shorten, egg-laying decreases, and the pupal stage shortens, which leads to a longer adult lifespan. The research determined the ideal environmental factors for each developmental stage of the beet armyworm, providing crucial insights into its adaptability in changing climates. These findings are important for predicting beet armyworm population dynamics and developing better pest management strategies.

## 1. Introduction

The increase in worldwide temperatures affects insects in numerous ways, including their survival, reproductive patterns, migration behaviors, and geographical distribution [1,2]. Climatic warming is also having negative effects on many insect species, leading to decreased fertility and increased mortality in overwintering species due to their poor adaptability to rapid climatic changes. In regions with warmer climates, insects experience faster developmental rates, leading to quicker population growth. This accelerated growth may contribute to their migration towards higher latitudes or altitudes [3]. Moreover, increasing temperatures enhance the reproduction and spread of specific pests, which intensifies the risk they pose to crops [4,5]. As a result, climate change could influence insect population patterns, especially by altering the distribution and frequency of pest outbreaks, which has significant consequences for both ecosystems and agriculture [6].

*Spodoptera exigua* (Lepidoptera: Noctuidae) is an extensively polyphagous pest that represents a major threat to numerous economically valuable crops in tropical and subtropical regions. This pest feeds on 170 plant species belonging to 35 different families, causing considerable damage [7]. Before the 1980s, *S. exigua* was mainly found in Beijing, Hebei, Henan, Shandong, and the Guanzhong area of Shaanxi Province. While it was also present in the Yangtze River Basin, as well as the Northeast and Northwest regions, its impact was relatively limited. However, by the late 1980s, the spread of *S. exigua* intensified, leading to more significant damage [8]. Along with damaging staple crops like corn, sorghum, and soybeans, *S. exigua* also causes considerable harm to economically important crops, such as vegetables, cotton, and sugar beets [9,10,11].

Climate change significantly affects the lifecycle, reproductive rate, distribution, behavior, and resistance of *S. exigua* to pesticides. Elevated temperatures could shorten its lifecycle, boost population density, and enable its spread into new regions, making pest management more challenging [12]. As a result, integrated pest management approaches are crucial, encompassing the optimization of agricultural planting patterns, strengthening biological control methods, and implementing accurate monitoring systems to address the challenges posed by climate change. Evaluating the effects of temperature fluctuations on *S. exigua* is vital for formulating effective control strategies.

Recent studies have concentrated on the biological traits, ecological aspects, artificial rearing, and breeding methods, as well as the migration behaviors of *S. exigua* [13,14,15]. While some research has explored the influence of temperature on *S. exigua* populations, most studies have focused on its effects on particular aspects of the pest’s biology [16,17]. This research employs meta-analysis to assess, in quantitative terms, the wider effects of temperature variations on *S. exigua* [18]. Meta-analysis is a structured research approach designed to combine and synthesize findings from several independent studies, allowing for more comprehensive and reliable conclusions [19]. Using meta-analysis, this study highlights the sensitivity of various biological traits of *S. exigua* to temperature variations under different conditions, providing valuable insights for the development of effective pest management strategies.

## 2. Materials and Methods

### 2.1. Literature Search and Selection Process

To comprehensively assess the impact of global climate change on the biological traits of *S. exigua*, this research performed a review of relevant literature from multiple databases. The search was carried out from September to October 2024, mainly using databases such as Web of Science, PubMed, Scopus, and CNKI. Furthermore, pertinent review papers were manually reviewed to identify studies that were not included in the database searches. The terms used in the search included “Beet armyworm” (also known as “Lesser armyworm” or “Spiny bollworm”), “climate change” (alternatively referred to as “global warming”), “temperature”, “precipitation”, “photoperiod”, and “biological characteristics” (including “life history traits”, “development”, “reproduction”, etc.).

The process of literature screening was divided into two stages: initially, studies unrelated to *S. exigua* or climate change were excluded based on their titles and abstracts; secondly, the full text of the remaining studies was reviewed to further exclude those that did not satisfy the inclusion criteria. The criteria for inclusion were as follows: (1) the study investigates how temperature variations influence the development time, lifespan, lifecycle, egg-laying period, fertility, and hatching rate of *S. exigua*. (2) The study includes data on additional variables, such as relative humidity, photoperiod, or other environmental factors. (3) The study provides experimental data, including sample size, mean values, standard errors, or standard deviations.

### 2.2. Data Extraction

Data pertinent to the study were collected from research articles that fulfilled the inclusion criteria. The main variables extracted were temperature (indicating various experimental temperature levels in °C), relative humidity, and biological traits (including developmental rate, generation time, and reproductive capacity, such as oviposition). When crucial statistical data, such as standard deviation or sample size, was not explicitly reported, the information was extracted from graphs using tools like WebPlotDigitizer (Version 4.7). For experiments involving multiple variables or datasets with several treatment groups, the results from each treatment were considered as independent effect sizes.

### 2.3. Statistical Analysis

In this research, the rma.mv function available in the “metafor” package of R version 4.3 was employed to perform the subsequent analyses [20]. Initially, we utilized a random-effects model to compute the relative risk (RR+) and we assessed the heterogeneity measure I² across studies using restricted maximum likelihood (REML) estimation. Depending on the value of I², explanatory variables that could affect the heterogeneity of effect sizes were then incorporated [21]. Next, a random-effects model was applied to assess the overall effect size across all treatment groups. Finally, statistical tests were performed to assess the mean effect size and the 95% confidence interval (CI), and the heterogeneity indices I² and the Q statistic (Qt) [22]. We performed separate meta-analyses for different independent variables to evaluate the response of *S. exigua* at various developmental stages to temperature, assessing the strength of these temperature effects.

In this meta-analysis, both random-effects and fixed-effects models were applied to integrate findings from multiple studies. Because of the significant heterogeneity observed across studies (e.g., variations in experimental locations, treatment procedures, etc.), the random-effects model was considered a more appropriate choice to address this variability [23]. We considered climate change-related factors (e.g., temperature and relative humidity) as independent variables and the biological traits of *S. exigua* as dependent variables. Effect sizes were computed by utilizing the Log Response Ratio (LRR), which compared the biological traits between the climate factor treatment groups and the control groups. Statistical significance was evaluated using 95% confidence intervals. To assess how different climate factors affect the biological traits of *S. exigua*, we performed subgroup analyses for temperature and relative humidity separately.

The Q statistic and I² index were used to assess the heterogeneity among the studies. The Q statistic tests for the presence of heterogeneity, while the I² value quantifies the degree of variability. An increased I² value signifies a higher degree of variability among the studies. The heterogeneity statistic is calculated by testing the weighted sum of squared deviations with k − 1 degrees of freedom, providing a measure of variability across studies. If the 95% confidence interval for the effect size includes zero, it suggests no significant difference between the experimental and control groups (*p* > 0.05). If the 95% confidence interval is entirely positive, it suggests a notably larger effect size in the experimental group than in the control group (*p* < 0.05). On the other hand, if the entire 95% confidence interval falls below zero, it suggests a significantly smaller effect size in the experimental group relative to the control group (*p* < 0.05) [24]. The decision to include explanatory variables was based on the significance of the cumulative effect size relative to zero and the *p*-value of the Qt statistic. The potential explanatory variables taken into account were the impacts of humidity and photoperiod on the overall effect size. Additionally, temperature data were considered as a continuous variable to assess their impact on the mean effect size. In the meta-analysis, the total heterogeneity was divided into between-group heterogeneity (variance attributed to categorical factors) and within-group heterogeneity (residual variance), with significance evaluated using a k − 1 test [25]. We also assessed potential publication bias by utilizing funnel plots and conducting Egger’s regression test [26]. If significant bias was detected, the “trim and fill” method was applied to correct for it. To assess publication bias, we analyzed the connection between effect size and sample size through the use of funnel plots. The presence of publication bias was determined based on the significance of the *p*-value [27]. If statistical significance persists after adjustment, this suggests that the findings are consistent and not affected by publication bias.

## 3. Results

### 3.1. Statistical Data

This research incorporates data from 33 different publications, encompassing a total of 264 individual observations. It examines 13 dependent variables related to temperature variation in *S. exigua*, including first-instar larvae (*n* = 20), second-instar larvae (*n* = 20), third-instar larvae (*n* = 20), fourth-instar larvae (*n* = 20), fifth-instar larvae (*n* = 20), sixth-instar larvae (*n* = 5), adult lifespan (*n* = 30), egg production (*n* = 38), larval period (*n* = 27), generation time (*n* = 11), pre-oviposition period (*n* = 5), average number of eggs per female (*n* = 16), and pupal period (*n* = 52) (Table 1).

### 3.2. Comprehensive Meta-Analyses with Random-Effects and Fixed-Effects Models

**Figure 1:**
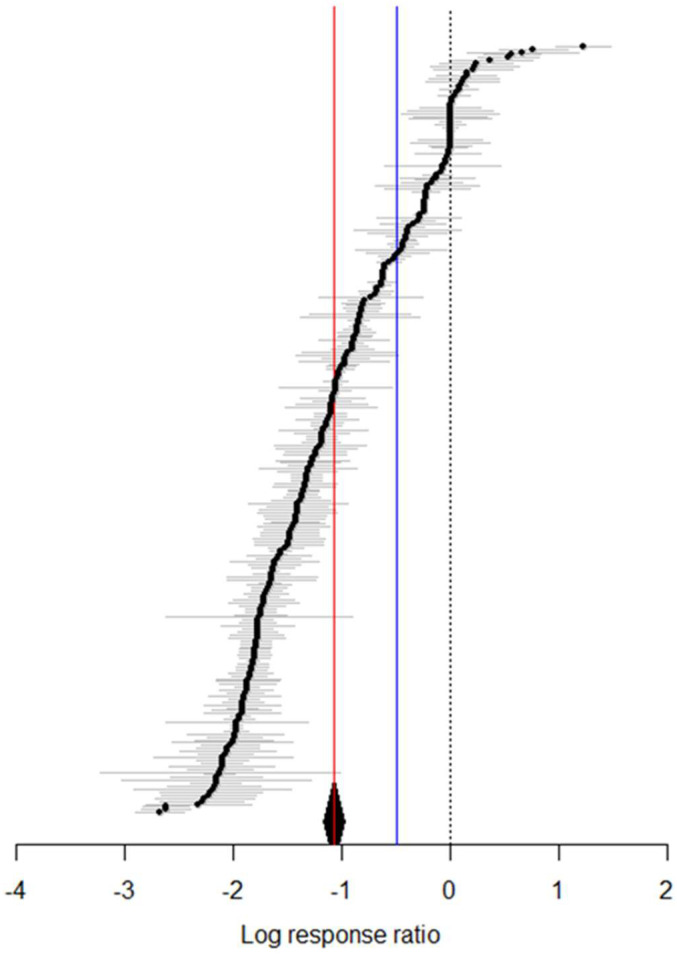
The forest plot shows the impact of temperature variation on *S. exigua*. The red line represents the results from the random-effects model, with a pooled effect size of −1.0733 and a 95% confidence interval from −1.1663 to −0.9803. The blue solid line indicates the results from the fixed-effects model, with a pooled effect size of −0.4942 and a 95% confidence interval ranging from −0.4985 to −0.4898. The black dashed line marks x = 0 and the gray line represents the standard error of individual factors.

**Figure 2:**
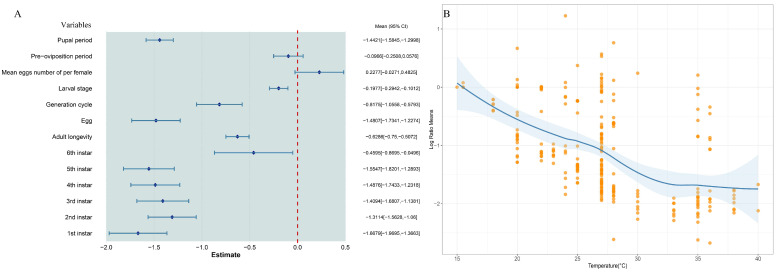
The impact of temperature variation on *S. exigua*. (**A**) illustrates the changes in biological traits of *S. exigua* as temperature increases. The red dashed line indicates x = 0. Dark blue squares represent the cumulative effect size for each temperature gradient, while the light blue line shows the 95% confidence interval. (**B**) depicts the temperature range curve for the optimal growth of *S. exigua*. The orange dots represents all factors and the blue solid line indicates the suitability of *S. exigua* to temperature variation. The shaded area indicates the 95% confidence interval.

**Figure 3:**
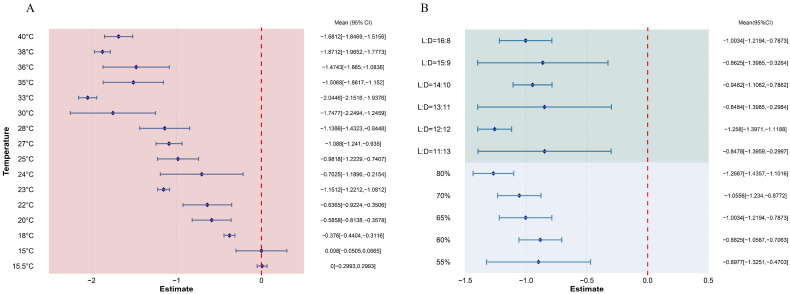
Forest plot showing the effects of temperature, photoperiod, and humidity on different biological indices of *S. exigua*. (**A**) illustrates how the physiological traits of *S. exigua* vary as the temperature increases. (**B**) depicts the response of *S. exigua* to changes in external environmental conditions. The dark blue squares indicate the cumulative effect size for each group, with the red dashed line representing x = 0. The light blue line marks the 95% confidence interval.

The findings indicate that elevated temperatures improved the adaptability of *S. exigua*, with a combined mean effect size of −1.0733 (95% CI: −1.1663, −0.9803; Figure 1). As temperatures increased, all dependent variables related to *S. exigua* significantly decreased, except for the average number of eggs per female and the pre-oviposition period, which remained unchanged (Figure 2A). When temperature was considered as a continuous variable, changes in *S. exigua* were observed across various temperature gradients (Figure 2B). Biological indicators of *S. exigua* significantly increased once temperatures exceeded 15 °C (Figure 3A). Biological activity peaked at 33 °C (Figure 3A), with a photoperiod of 12:12 and humidity at 80% (Figure 3B).

### 3.3. The Effect of Temperature on Developmental Duration

**Figure 4:**
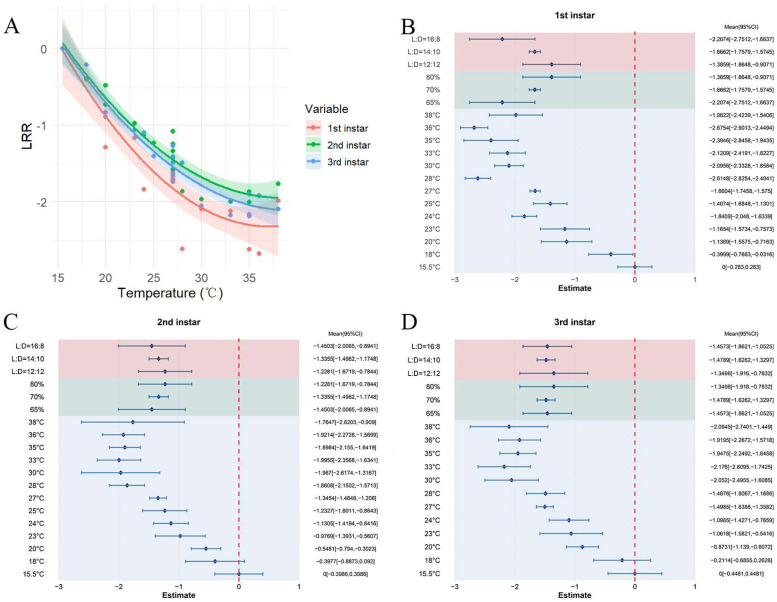
The impact of temperature on the developmental stages of *S. exigua*. (**A**) displays the variations in developmental time for first to third instar larvae at different temperature levels. (**B**–**D**) depict the reaction of first to third instar larvae to changes in external environmental conditions.

In all the studies examined, higher temperatures were associated with a decrease in the developmental period of first-instar *S. exigua*, resulting in a combined mean effect size of −1.6678 (CI: −1.9695, −1.3663; Figure S1). Within the temperature range of 15.5–38 °C, the developmental time of first-instar *S. exigua* decreased significantly as temperature increased (Figure 4A). Both the random-effects and fixed-effects models showed Q (df = 19) = 462.6090, *p* < 0.0001; this suggests that the variability across studies significantly influenced the cumulative effect size, highlighting the need to include explanatory variables (Figure 4B). The findings revealed that humidity (Qm = 5.1338, *p* = 0.0235) influences the cumulative effect size and that variations in relative humidity and photoperiod have distinct effects on the relationship between temperature variation and the developmental period of first-instar *S. exigua*. The optimal conditions for first-instar development were identified as 36 °C, 65% relative humidity, and a photoperiod of 16:8.

In all experiments, elevated temperatures led to a reduced developmental period for second-instar *S. exigua*, the pooled mean effect size was −1.3114 (CI: −1.5628, −1.0600; Figure S2). The developmental period of second-instar *S. exigua* decreased significantly with increasing temperature within the 15.5–38 °C range (Figure 4A). The findings from both random-effects and fixed-effects models indicated a significant between-study heterogeneity (Q (df = 19) = 167.9342, *p* < 0.0001), influencing the cumulative effect size, which underscores the necessity of incorporating explanatory variables (Figure 4C). These findings suggest that the optimal conditions for second-instar *S. exigua* development are 33 °C, 65% relative humidity, and a photoperiod of 16:8.

Across all studies, elevated temperatures resulted in a shortened developmental period for third-instar *S. exigua*, with a pooled mean effect size of −1.4094 (CI: −1.6807, −1.1381; Figure S3). Within the temperature range of 15.5–38 °C, the developmental time of third-instar *S. exigua* decreased significantly as temperature increased (Figure 4A). Results from both random-effects and fixed-effects models showed Q (df = 18) = 134.8382, *p* < 0.0001; this suggests significant variability between studies that affected the cumulative effect size, emphasizing the importance of incorporating explanatory variables (Figure 4C). These findings suggest that the optimal conditions for third-instar *S. exigua* development are 33 °C, 70% relative humidity, and a photoperiod of 14:10.

**Figure 5:**
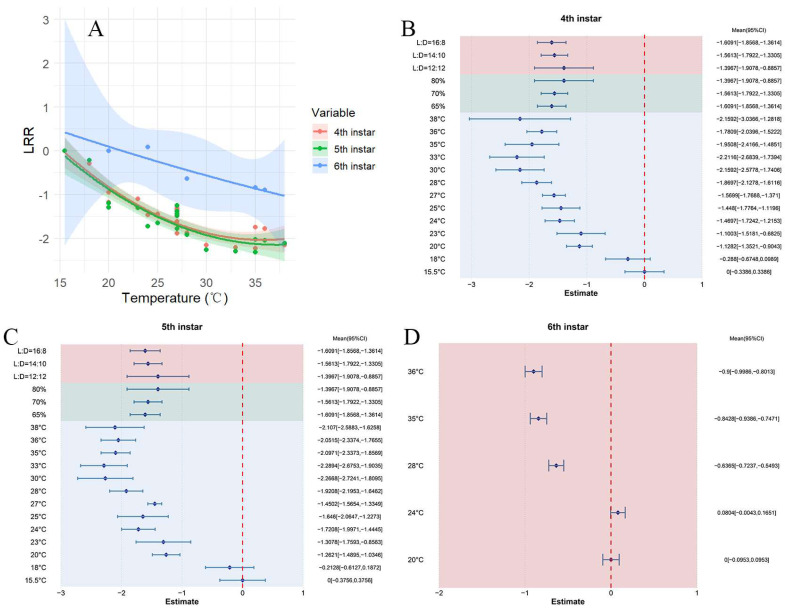
The impact of temperature on the developmental stages of *S. exigua*. (**A**) illustrates how developmental time for fourth to sixth instar larvae changes across varying temperatures. (**B**–**D**) represent the responses of fourth to sixth instar larvae to different external environmental conditions.

Across the studies, elevated temperatures resulted in a shorter developmental period for fourth-instar *S. exigua*, with a combined mean effect size of −1.4876 (CI: −1.7433, −1.2318; Figure S4). Within the temperature span of 15.5–38 °C, the developmental period of fourth-instar *S. exigua* significantly shortened with rising temperatures (Figure 5A). Both random-effects and fixed-effects model results revealed Q (df = 19) = 192.8081, *p* < 0.0001, suggesting substantial heterogeneity between studies that affected the cumulative effect size, emphasizing the need for explanatory variables to account for this variability (Figure 5B). These findings suggest that the optimal conditions for fourth-instar *S. exigua* development are 33 °C, 65% relative humidity, and a photoperiod of 16:8.

Across all studies, elevated temperatures led to a reduction in the developmental period of fifth-instar *S. exigua*, with a combined mean effect size of −1.5547 (CI: −1.8201, −1.2893; Figure S5). Within the 15.5–38 °C temperature range, the developmental time of fifth-instar *S. exigua* significantly decreased as the temperature rose (Figure 5A). The results from both random-effects and fixed-effects models indicated Q (df = 19) = 195.3696, *p* < 0.0001, highlighting considerable variability between studies that impacted the overall effect size, thus requiring the incorporation of explanatory variables (Figure 5C). The results indicate that both humidity and photoperiod have an impact on the overall effect size, with variations in these environmental factors affecting the developmental period of fifth-instar *S. exigua* in distinct ways. The optimal conditions for fifth-instar development were identified as 33 °C, 65% relative humidity, and a photoperiod of 16:8.

Across all studies, elevated temperatures resulted in a shortened developmental period for sixth-instar *S. exigua*, with a pooled mean effect size of −0.4334 (CI: −0.4744, −0.3923; Figure S6). Within the temperature range of 20–36 °C, the developmental duration of sixth-instar *S. exigua* significantly decreased as temperature increased (Figure 5A). The optimal condition for sixth-instar development was identified as 36 °C (Figure 5D).

**Figure 6:**
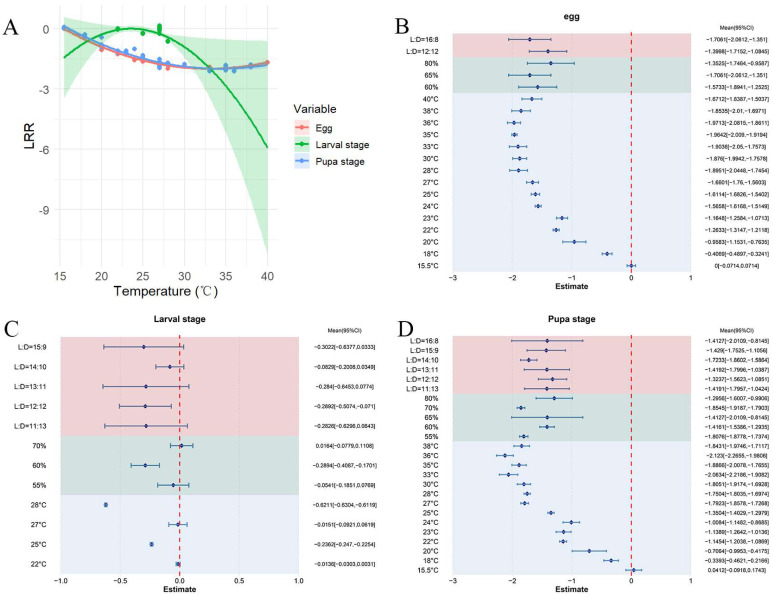
The effect of temperature on *S. exigua* egg, larval, and pupal periods. (**A**) shows the changes in developmental time for eggs, larvae, and pupae across different temperatures. (**B**–**D**) illustrate the response of eggs, larvae, and pupae to varying external environmental conditions.

Across all studies, higher temperatures led to a shortened developmental period for *S. exigua* eggs and the pooled mean effect size was calculated as −1.4807 (CI: −1.7341, −1.2274; Figure S7). The developmental time of *S. exigua* eggs increased initially and then decreased as the temperature rose within the range of 15.5–40 °C (Figure 6A). The results from both the random-effects and fixed-effects models showed Q (df = 18) = 4085.3176, *p* < 0.0001, suggesting that variations between studies had a substantial effect on the cumulative effect size, highlighting the need to include explanatory factors like humidity and photoperiod (Figure 6B). The ideal conditions for the development of *S. exigua* eggs were determined to be 36 °C, 65% relative humidity, and a 16:8 light/dark photoperiod. In all studies, elevated temperatures led to a reduction in the larval duration of *S. exigua*, with an overall mean effect size of −0.0997 (CI: −0.2942, −0.1012; Figure S8). Within the 22–28 °C temperature range, as the temperature rose, the larval duration of *S. exigua* was reduced (Figure 6A). The results from both the random-effects and fixed-effects models indicated Q (df = 26) = 9635.8421, *p* < 0.0001, suggesting considerable heterogeneity across studies, which affected the cumulative effect size. This highlights the necessity of incorporating explanatory variables (Figure 6C). The cumulative effect size was influenced by both photoperiod and relative humidity, with each factor impacting the larval period of *S. exigua* to different extents. The longest larval duration occurred at 28 °C, 60% relative humidity, and a 12:12 photoperiod.

In all studies, elevated temperatures resulted in a reduced pupal period for *S. exigua*, with a pooled mean effect size of −1.4421 (CI: −1.5845, −1.2998; Figure S9). Within the 15.5–38 °C temperature range, the pupal period duration significantly shortened with rising temperatures (Figure 6A). The results from both random-effects and fixed-effects models revealed Q (df = 51) = 1928.5778, *p* < 0.0001, suggesting that variations between studies impacted the cumulative effect size, emphasizing the need for explanatory variables (Figure 6D). The results indicate that relative humidity and photoperiod are key factors influencing the cumulative effect size, with different humidity levels exerting varying effects on the pupal duration of *S. exigua*. The shortest pupal developmental period was observed at 36 °C, with 70% relative humidity and a photoperiod of 14:10.

### 3.4. The Influence of Temperature on the Ovipositional Behavior of Female Adults

**Figure 7:**
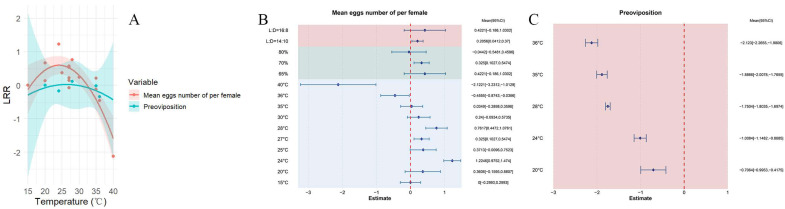
The effect of temperature on the ovipositional behavior and pre-oviposition period of female *S. exigua* adults. (**A**) shows how ovipositional behavior and the pre-oviposition period of female adults change with varying temperature. (**B**,**C**) illustrate the response of ovipositional behavior and the pre-oviposition period to different external environmental conditions.

Across all analyses, elevated temperatures were found to decrease the average egg-laying capacity of female *S. exigua*, with a pooled mean effect size of 0.2277 (CI: −0.0271, −0.4825; Figure S10). Across the temperature range of 15.5–40 °C, the egg-laying capacity of female *S. exigua* initially increased and then decreased as the temperature rose (Figure 7A). Results from both the random-effects and fixed-effects models showed Q (df = 15) = 113.0038, *p* < 0.0001, revealing significant heterogeneity between studies that affected the cumulative effect size, thereby necessitating the inclusion of explanatory variables (Figure 7B). The maximum egg-laying capacity was recorded at 24 °C, 70% relative humidity, and a photoperiod of 14:10.

Across all studies, higher temperatures were found to reduce the pre-oviposition period of female *S. exigua*, with a pooled mean effect size of −0.0966 (CI: −0.2508, −0.0576; Figure S11). Within the temperature span of 20–36 °C, the pre-oviposition period initially increased and then decreased as temperature rose (Figure 7A). The shortest pre-oviposition period for adult female *S. exigua* was observed at 36 °C (Figure 7C).

### 3.5. The Effect of Temperature on Generation Time and Adult Lifespan

**Figure 8:**
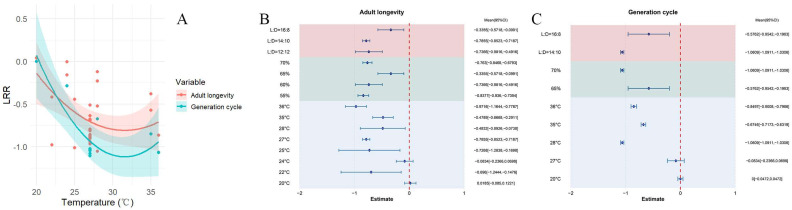
The effect of temperature fluctuations on the lifespan of *S. exigua* is presented as follows: (**A**) demonstrates the changes in the adult lifespan and total lifecycle duration with increasing temperature. (**B**) illustrates how the adult lifespan varies in response to changes in external environmental factors. (**C**) depicts the influence of environmental changes on the lifecycle duration of *S. exigua*.

Across all the studies, higher temperatures were associated with a reduction in the adult lifespan of *S. exigua*, yielding a combined mean effect size of −0.6286 (CI: −0.7500, −0.5072; Figure S12). Throughout the temperature spectrum of 10–41 °C, the lifespan of adult *S. exigua* showed a significant reduction with rising temperatures (Figure 8A). Both the random-effects and fixed-effects models revealed substantial heterogeneity across studies (Q (df = 29) = 616.2937, *p* < 0.0001), which impacted the cumulative effect size and necessitated the inclusion of explanatory variables (Figure 8B). It is important to note that the photoperiod (QM = 17.4313, *p* = 0.0002) significantly influenced the cumulative effect size, showing differing effects on the lifespan of adult *S. exigua*. The shortest lifespan occurred at 36 °C, with 55% relative humidity and a 14:10 light-dark photoperiod.

Across the various studies, rising temperatures led to a shortened lifecycle of *S. exigua*, with a pooled mean effect size of −0.8175 (CI: −1.0058, −0.5793; Figure S13). Within the temperature span of 20 to 36 °C, the lifecycle of *S. exigua* consistently shortened as the temperature increased (Figure 8A). Results from both the random-effects and fixed-effects models indicated Q (df = 9) = 2513.7723, *p* < 0.0001, revealing significant heterogeneity among the studies, which affects the overall effect size and requires the inclusion of explanatory variables (Figure 8C). Notably, the photoperiod (QM = 6.2278, *p* = 0.0126) and relative humidity (QM = 6.2278, *p* = 0.0126) are identified as factors that affect the cumulative effect size, with varying degrees of influence on the lifecycle of *S. exigua*. The shortest lifecycle was recorded at 36 °C, with 70% relative humidity and a 14:10 light-dark ratio.

### 3.6. Model Validation

**Figure 9:**
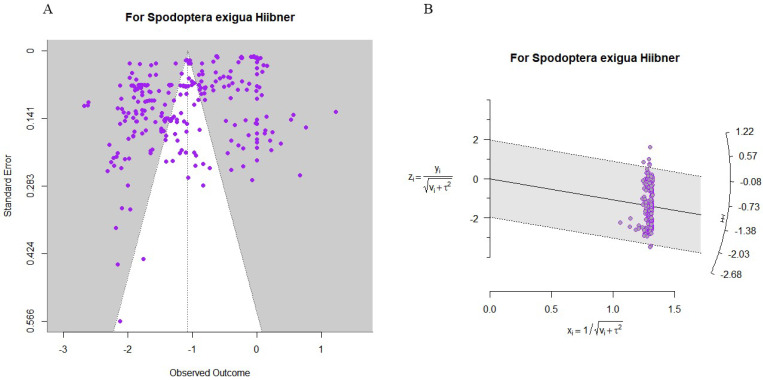
(**A**) Funnel plot; (**B**) radar plot.

We assessed the possibility of publication bias using both funnel and radar charts, and assessed the robustness of our results by calculating the fail-safe N. The findings from the funnel plot (Figure 9A; z = 3.6893, *p* = 0.0002), radar plot (Figure 9B), and fail-safe N (N = 27,385) all supported the reliability of our conclusions.

## 4. Discussion

In the context of global warming, it is expected that the distribution of *S. exigua* will significantly expand. To assess its adaptability to higher temperatures, this study conducted a comprehensive meta-analysis of 33 relevant publications. Through a rigorous literature selection and data extraction process, we quantified the variation in *S. exigua*’s responses under different temperature conditions. The results show that the adaptability of *S. exigua* improves progressively with increasing temperatures, with a marked enhancement observed particularly between 15 °C and 40 °C [28,29]. At 33 °C, *S. exigua* reaches its peak adaptability, a finding that is consistent with previous studies and reinforces the reliability of the analytical model used [30,31]. Generally, a longer lifespan in insects tends to be associated with an extended reproductive period, offering more opportunities for egg laying. However, under the environmental conditions at 33 °C, the adult lifespan is reduced, leading to a shorter reproductive cycle, which in turn decreases egg production and results in lower fecundity.

Given the significant potential economic impact that *S. exigua* may have on agricultural production, monitoring its adaptability is crucial. Elevated temperatures generally expedite the developmental processes of insects, with growth rates strongly influenced by environmental conditions. Higher temperatures can shorten the developmental periods of various life stages—eggs, larvae, pupae, and adults [32,33]. Consequently, higher temperatures are anticipated to shorten the total lifecycle duration of *S. exigua*. Through meta-analysis, this study indicates that the species shows peak adaptability at temperatures around 33 °C. Given the significance of early warning systems for tracking insect invasion routes and identifying appropriate environmental conditions, the results offer essential information to guide future monitoring and alert strategies.

As ectothermic organisms, insect populations are highly influenced by temperature, which plays a central role in their distribution patterns. With the accelerating effects of global warming, many insect species have experienced shifts in their geographical ranges, leading to a broader distribution [34,35,36]. Temperature is a crucial factor affecting the survival, development, and reproduction of *S. exigua*. Studies have shown that an optimal temperature range can significantly promote its growth and development, while temperature fluctuations can disrupt its growth cycle and reduce reproductive capacity. To investigate this connection in more detail, we analyzed the effect of temperature on different developmental stages of *S. exigua* and created the corresponding response curves. The results reveal that temperature affects each developmental stage differently, with distinct responses observed at each stage of growth.

The continuous rise in global temperatures is expected to significantly affect ecosystem function and structure, resulting in shifts in the distribution of biological habitats [37,38,39]. Climate change is expected to increase both the frequency and geographic spread of insect infestations, leading to greater agricultural damage. It is expected that the adaptability of *S. exigua* will enhance over time under future warming scenarios. The findings of this study offer important insights for policymakers to formulate more effective pest management strategies. These strategies will be essential in mitigating the potential economic losses that climate change may inflict on agricultural production in the future.

## 5. Conclusions

*Spodoptera exigua* is a significant agricultural pest that poses a serious threat to a wide range of crops. As such, developing an effective prediction model to monitor its presence in the field is essential. Conventional prediction approaches frequently use developmental parameters that depend on temperature and models of thermal biology to forecast changes in insect phenology. This study evaluated the adaptability of *S. exigua* to various temperature conditions by synthesizing data from 264 temperature-dependent experiments and performing a meta-analysis to predict its optimal temperature range for survival. Our results indicate that the adaptability of *S. exigua* improves as temperatures rise within the range of 15–40 °C, with optimal growth conditions occurring between 30 and 35 °C. Although the distribution of *S. exigua* has expanded over recent decades, current management strategies remain inadequate. It is essential to emphasize the significance of prompt chemical control. By predicting the optimal temperature range for *S. exigua* and preparing the appropriate chemical agents in advance, pest control efforts can be more effective, reducing its impact on agricultural production.

## Figures and Tables

**Table 1:** Dataset on biological indicators of *S. exigua* (TR stands for temperature range, CT stands for controlled temperature, N represents sample size, and V stands for variable.).

TR	CT	N	V
15.5–38 °C	15.5	20	First instar
15.5–38 °C	15.5	20	Second instar
15.5–38 °C	15.5	20	Third instar
15.5–38 °C	15.5	20	Fourth instar
15.5–38 °C	15.5	20	Fifth instar
20–36 °C	20	5	Sixth instar
20–36 °C	20	30	Adult longevity
15.5–40 °C	15.5	19	Egg
20–36 °C	20	10	Generation cycle
22–28 °C	22	27	Larval stage
15–40 °C	15	16	Mean eggs number of per female
20–36 °C	20	5	Pre-oviposition
15.5–38 °C	15.5	52	Pupa stage
15–40 °C	15.5	264	*S. exigua*

## Data Availability

The data that support the findings can be accessed in a public repository at: https://doi.org/10.6084/m9.figshare.28057121.v2 (accessed on 19 December 2024).

## Supplementary Materials

The following supporting information can be downloaded at: https://www.mdpi.com/article/10.3390/insects16020155/s1, Figure S1: Comparison of effect sizes between models using random-effects and fixed-effects approaches; Figure S2: Comparison of effect sizes between models using random-effects and fixed-effects approaches; Figure S3: Comparison of effect sizes between models using random-effects and fixed-effects approaches; Figure S4: Comparison of effect sizes between models using random-effects and fixed-effects approaches; Figure S5: Comparison of effect sizes between models using random-effects and fixed-effects approaches; Figure S6: Comparison of effect sizes between models using random-effects and fixed-effects approaches; Figure S7: Comparison of effect sizes between models using random-effects and fixed-effects approaches; Figure S8: Comparison of effect sizes between models using random-effects and fixed-effects approaches; Figure S9: Comparison of effect sizes between models using random-effects and fixed-effects approaches; Figure S10: Comparison of effect sizes between models using random-effects and fixed-effects approaches; Figure S11: Comparison of effect sizes between models using random-effects and fixed-effects approaches; Figure S12: Comparison of effect sizes between models using random-effects and fixed-effects approaches; Figure S13: Comparison of effect sizes between models using random-effects and fixed-effects approaches.
