# Supplementary material for: Impact of Temperature Variation on the Biological Traits and Lifecycle of Spodoptera exigua (Lepidoptera: Noctuidae): A Meta-Analysis Approach
Source: Insects. 2025 Feb 3;16(2):155. doi: 10.3390/insects16020155 (PMC11855858; doi:10.3390/insects16020155)
Supplement: Supplementary file 1 [file insects-16-00155-s001.zip › insects-3408857-supplementary.pdf]

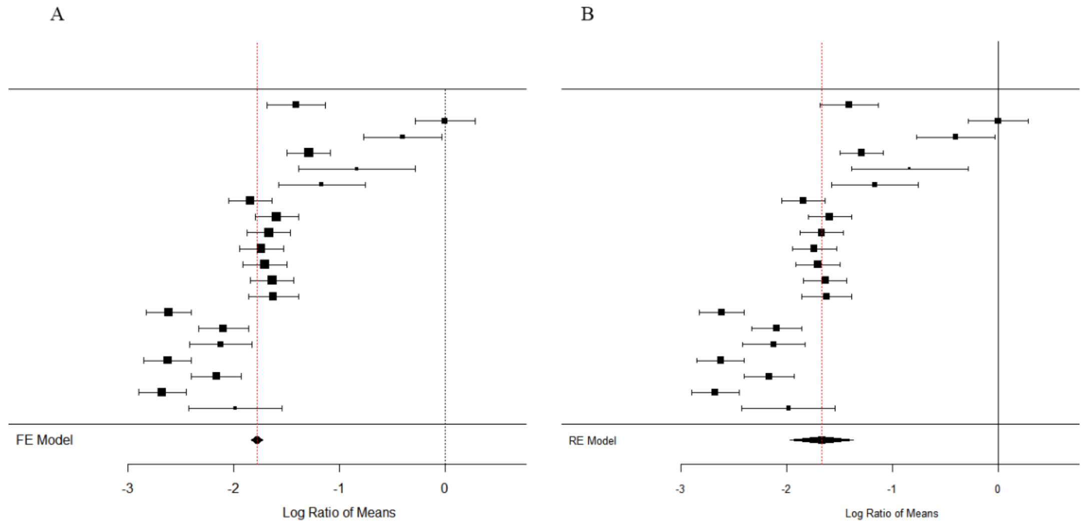

Figure S1 Comparison of effect sizes between the random-effects model and fixed-effects model (Panel A shows the results from the fixed-effects model, E = -1.7787, 95% confidence interval from -1.8328 to -1.7245, Q (df=19) = 462.6090, $p < 0.0001$, I^2 = 95.89%. Panel B shows the results from the random-effects model, E = -1.6679, 95% confidence interval from -1.9695 to -1.3663, Q (df=19) = 462.6090, $p < 0.0001$, I^2 = 96.71%). The results indicate that, under both the random-effects and fixed-effects models, the first instar larval development time of *Spodoptera exigua* significantly shortens with increasing temperature. The black squares represent the cumulative effect size for each variable, and the black horizontal lines represent the 95% confidence intervals. In both Panel A and Panel B, the red line indicates the cumulative effect size.

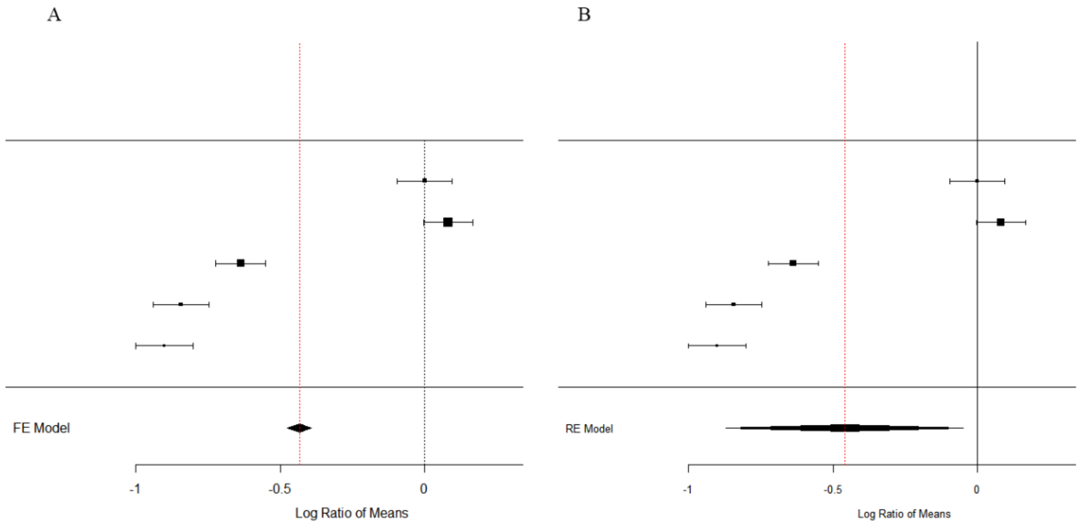

Figure S2 Comparison of effect sizes between the random-effects model and fixed-effects model (Panel A shows the results from the fixed-effects model, E = -1.3156, 95% confidence interval from -1.3156 to -1.2378, Q (df=19) = 167.9342, p < 0.0001, I^2 = 88.69%. Panel B shows the results from the random-effects model, E = -1.3114, 95% confidence interval from -1.5628 to -1.0600, Q (df=19) = 167.9342, p < 0.0001, I^2 = 90.00%). The results indicate that, under both the random-effects and fixed-effects models, the second instar larval development time of *Spodoptera exigua* significantly shortens with increasing temperature. The black squares represent the cumulative effect size for each variable, and the black horizontal lines represent the 95% confidence intervals. In both Panel A and Panel B, the red line indicates the cumulative effect size.

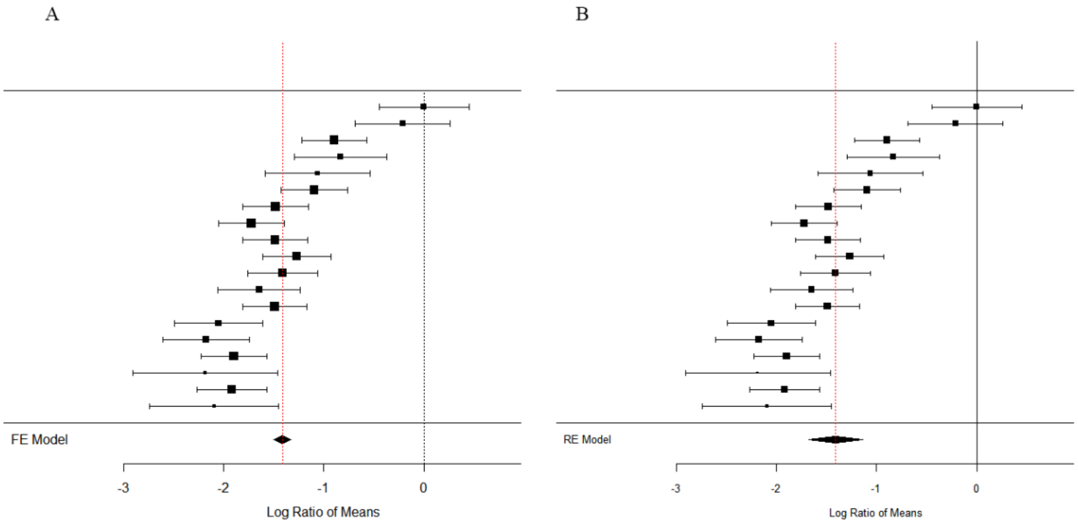

Figure S3 Comparison of effect sizes between the random-effects model and fixed-effects model (Panel A shows the results from the fixed-effects model, E = -1.4120, 95% confidence interval from -1.5000 to -1.3240, Q (df = 18) = 134.8382, $p < 0.0001$, I^2 = 86.65%. Panel B shows the results from the random-effects model, E = -1.4094, 95% confidence interval from -1.6807 to -1.1381, Q (df = 18) = 134.8382, $p < 0.0001$, I^2 = 89.17%). The results indicate that, under both the random-effects and fixed-effects models, the third instar larval development time of *Spodoptera exigua* significantly shortens with increasing temperature. The black squares represent the cumulative effect size for each variable, and the black horizontal lines represent the 95% confidence intervals. In both Panel A and Panel B, the red line indicates the cumulative effect size.

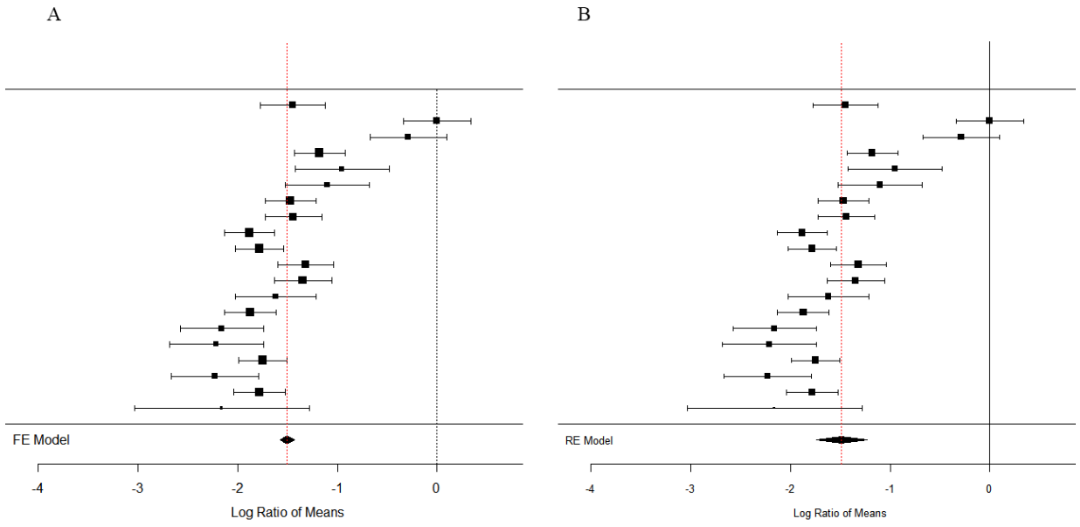

Figure S4 Comparison of effect sizes between the random-effects model and fixed-effects model (Panel A shows the results from the fixed-effects model, E = -1.5018, 95% confidence interval from -1.5714 to -1.4321, Q (df = 19) = 192.8081, $p < 0.0001$, I^2 = 90.15%. Panel B shows the results from the random-effects model, E = -1.4876, 95% confidence interval from -1.7433 to -1.2318, Q (df = 19) = 192.8081, $p < 0.0001$, I^2 = 92.27%). The results indicate that, under both the random-effects and fixed-effects models, the fourth instar larval development time of *Spodoptera exigua* significantly shortens with increasing temperature. The black squares represent the cumulative effect size for each variable, and the black horizontal lines represent the 95% confidence intervals. In both Panel A and Panel B, the red line indicates the cumulative effect size.

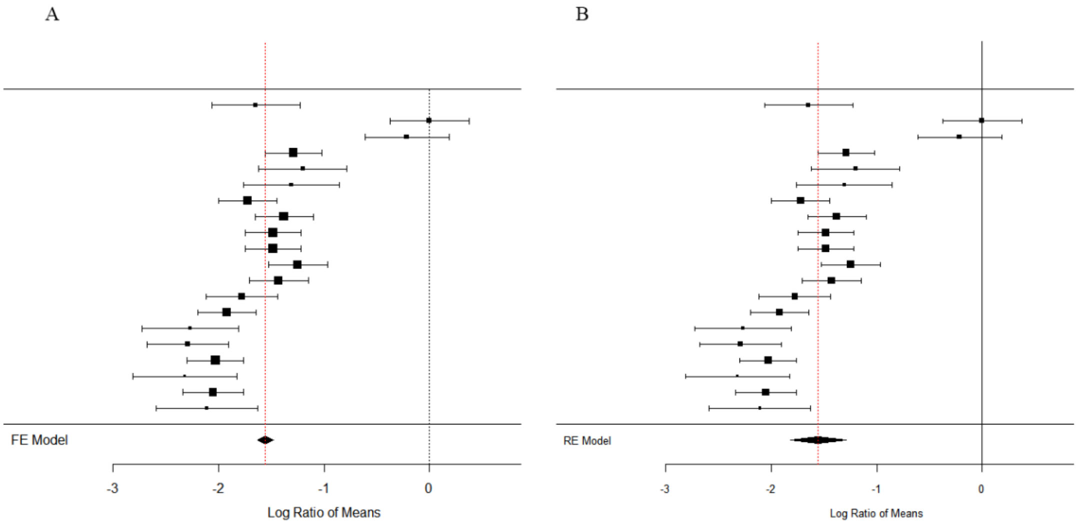

Figure S5 Comparison of effect sizes between the random-effects model and fixed-effects model (Panel A shows the results from the fixed-effects model, E = -1.5542, 95% confidence interval from -1.6265 to -1.4818, Q (df = 19) = 195.3696, $p < 0.0001$, I^2 = 90.27%. Panel B shows the results from the random-effects model, E = -1.5547, 95% confidence interval from -1.8201 to -1.2893, Q (df = 19) = 195.3696, $p < 0.0001$, I^2 = 92.39%). The results indicate that, under both the random-effects and fixed-effects models, the fifth instar larval development time of *Spodoptera exigua* significantly shortens with increasing temperature. The black squares represent the cumulative effect size for each variable, and the black horizontal lines represent the 95% confidence intervals. In both Panel A and Panel B, the red line indicates the cumulative effect size.

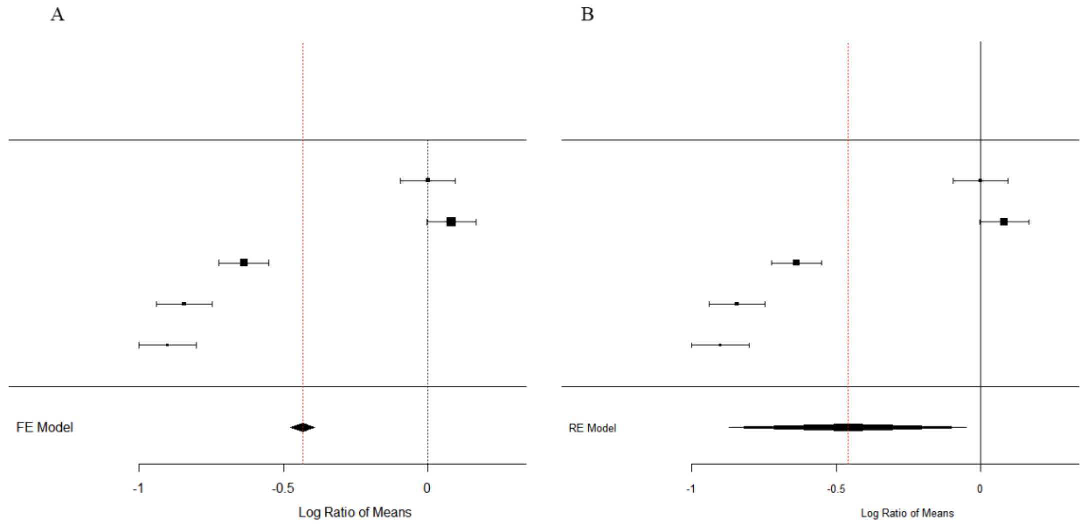

Figure S6 Comparison of effect sizes between the random-effects model and fixed-effects model (Panel A shows the results from the fixed-effects model, E = -0.4334, 95% confidence interval from -0.4744 to -0.3923, Q (df = 4) = 397.6805, $p < 0.0001$, I^2 = 98.99%. Panel B shows the results from the random-effects model, E = -0.4595, 95% confidence interval from -0.8695 to -0.0496, Q (df = 4) = 397.6805, $p < 0.0001$, I^2 = 98.99%). The results indicate that, under both the random-effects and fixed-effects models, the sixth instar larval development time of *Spodoptera exigua* significantly shortens with increasing temperature. The black squares represent the cumulative effect size for each variable, and the black horizontal lines represent the 95% confidence intervals. In both Panel A and Panel B, the red line indicates the cumulative effect size.

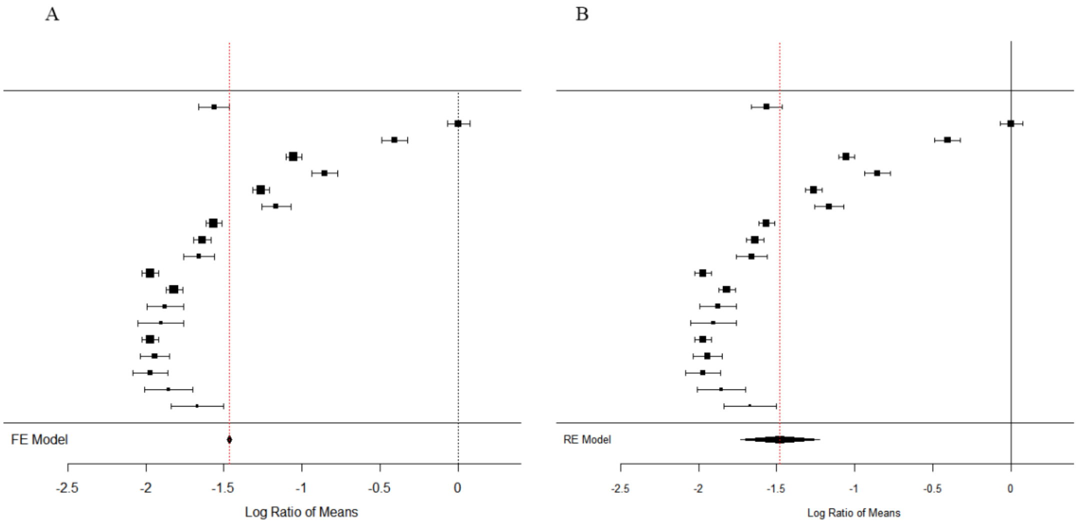

Figure S7 Comparison of effect sizes between the random-effects model and fixed-effects model (Panel A shows the results from the fixed-effects model, E = -1.4641, 95% confidence interval from -1.4804 to -1.4478, Q (df = 18) = 4085.3176, $p < 0.0001$, I^2 = 98.57%. Panel B shows the results from the random-effects model, E = -1.4807, 95% confidence interval from -1.7341 to -1.2274, Q (df = 18) = 4085.3176, $p < 0.0001$, I^2 = 98.57%). The results indicate that, under both the random-effects and fixed-effects models, the egg stage development time of *Spodoptera exigua* significantly shortens with increasing temperature. The black squares represent the cumulative effect size for each variable, and the black horizontal lines represent the 95% confidence intervals. In both Panel A and Panel B, the red line indicates the cumulative effect size.

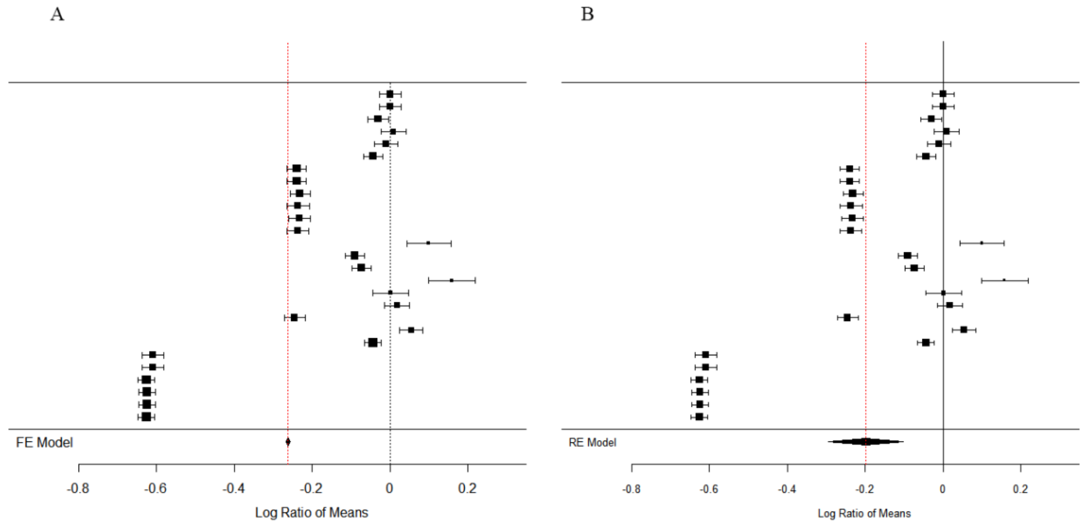

Figure S8 Comparison of effect sizes between the random-effects model and fixed-effects model (Panel A shows the results from the fixed-effects model, E = -0.2662, 95% confidence interval from -0.2674 to -0.2570, Q (df = 26) = 9635.8421, $p < 0.0001$, I^2 = 99.73%. Panel B shows the results from the random-effects model, E = -0.1977, 95% confidence interval from -0.2942 to -0.1012, Q (df = 26) = 9635.8421, $p < 0.0001$, I^2 = 99.71%). The results indicate that, under both the random-effects and fixed-effects models, the larval stage development time of *Spodoptera exigua* significantly shortens with increasing temperature. The black squares represent the cumulative effect size for each variable, and the black horizontal lines represent the 95% confidence intervals. In both Panel A and Panel B, the red line indicates the cumulative effect size.

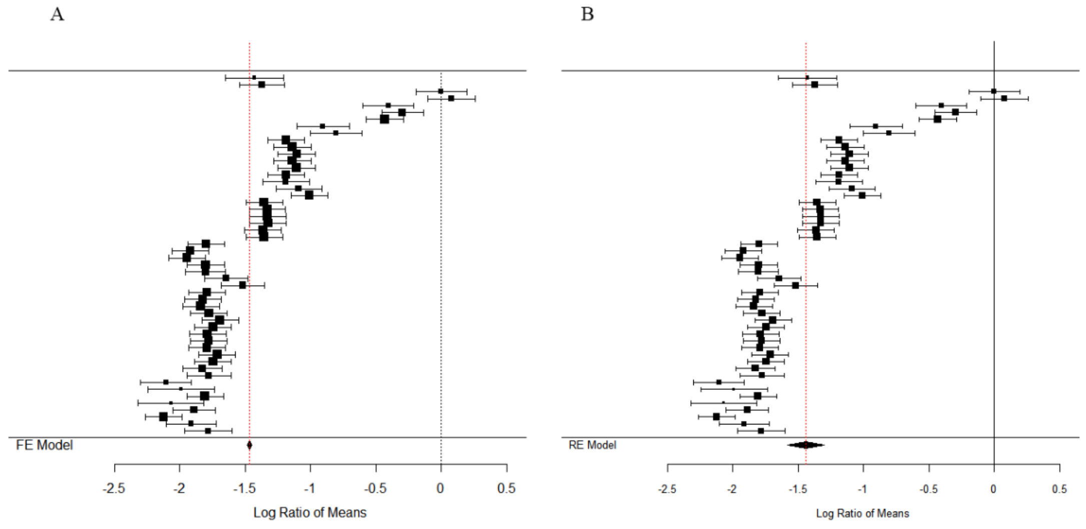

Figure S9 Comparison of effect sizes between the random-effects model and fixed-effects model (Panel A shows the results from the fixed-effects model, E = -0.4660, 95% confidence interval from -1.4873 to -1.4447, Q (df = 51) = 1928.5788, $p < 0.0001$, I^2 = 97.36%. Panel B shows the results from the random-effects model, E = -1.4421, 95% confidence interval from -1.5845 to -1.2998, Q (df = 51) = 1928.5788, $p < 0.0001$, I^2 = 97.75%). The results indicate that, under both the random-effects and fixed-effects models, the pupal stage development time of *Spodoptera exigua* significantly shortens with increasing temperature. The black squares represent the cumulative effect size for each variable, and the black horizontal lines represent the 95% confidence intervals. In both Panel A and Panel B, the red line indicates the cumulative effect size.

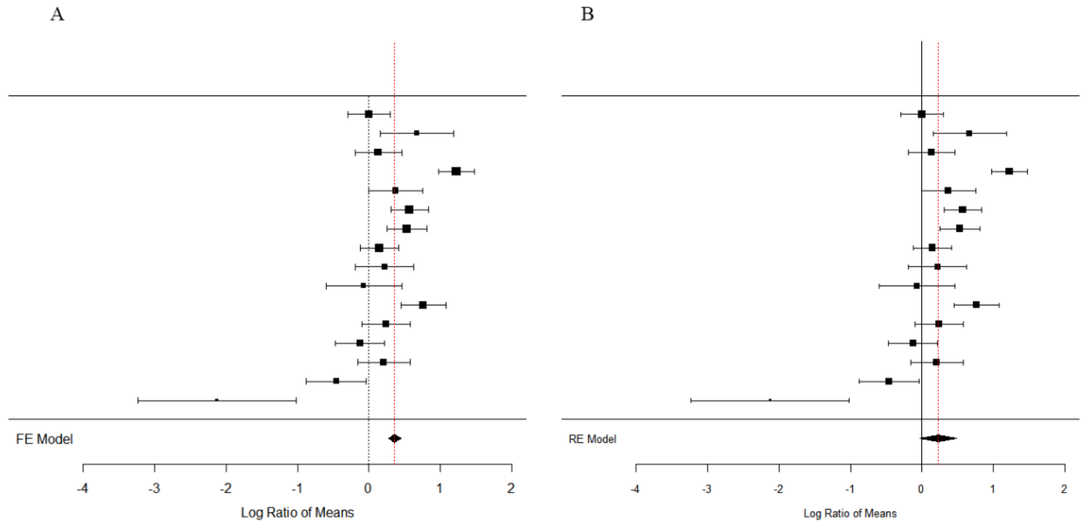

Figure S10 Comparison of effect sizes between the random-effects model and fixed-effects model (Panel A shows the results from the fixed-effects model, E = 0.3597, 95% confidence interval from 0.2750 to 0.4445, Q (df = 15) = 113.0038, $p < 0.0001$, I^2 = 86.73%. Panel B shows the results from the random-effects model, E = 0.2277, 95% confidence interval from -0.0271 to 0.4825, Q (df = 15) = 113.0038, $p < 0.0001$, I^2 = 88.27%). The results indicate that, under both the random-effects and fixed-effects models, the female average fecundity of *Spodoptera exigua* significantly shortens with increasing temperature. The black squares represent the cumulative effect size for each variable, and the black horizontal lines represent the 95% confidence intervals. In both Panel A and Panel B, the red line indicates the cumulative effect size.

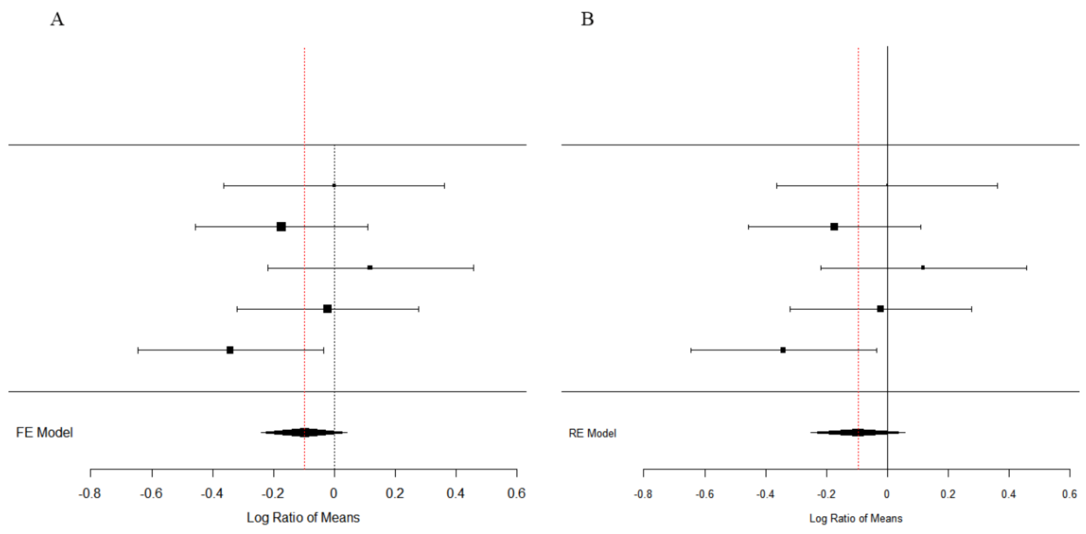

Figure S11 Comparison of effect sizes between the random-effects model and fixed-effects model (Panel A shows the results from the fixed-effects model, E = -0.0990, 95% confidence interval from -0.2392 to 0.0412, Q (df = 4) = 4.8222, $p < 0.0001$, I^2 = 17.05%. Panel B shows the results from the random-effects model, E = -0.0966, 95% confidence interval from -0.2508 to 0.0576, Q (df = 4) = 4.8222, $p < 0.0001$, I^2 = 16.81%). The results indicate that, under both the random-effects and fixed-effects models, the pre-oviposition period of *Spodoptera exigua* significantly shortens with increasing temperature. The black squares represent the cumulative effect size for each variable, and the black horizontal lines represent the 95% confidence intervals. In both Panel A and Panel B, the red line indicates the cumulative effect size.

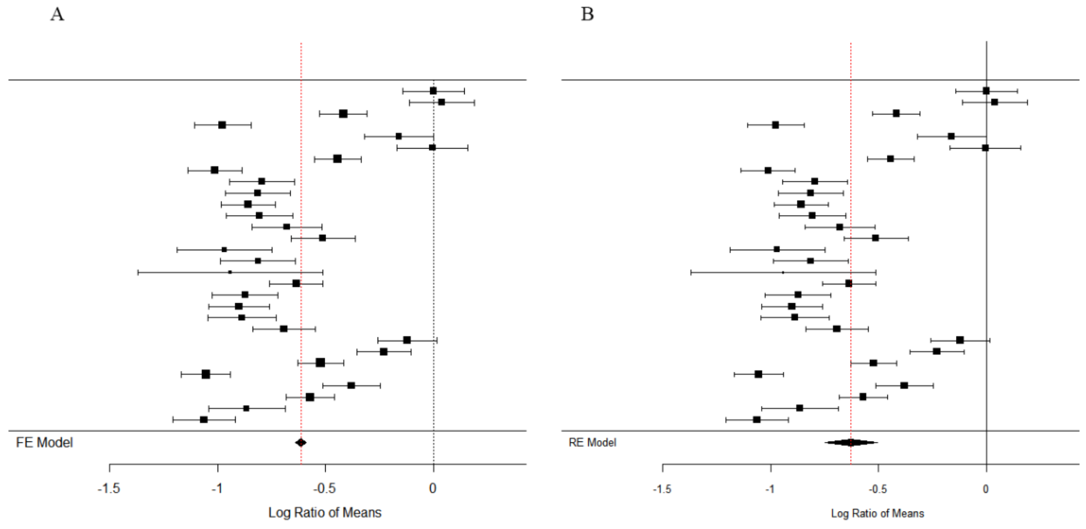

Figure S12 Comparison of effect sizes between the random-effects model and fixed-effects model (Panel A shows the results from the fixed-effects model, E = -0.6143, 95% confidence interval from -0.6397 to -0.5888, Q (df = 29) = 616.2937, $p < 0.0001$, I^2 = 95.29%. Panel B shows the results from the random-effects model, E = -0.6286, 95% confidence interval from -0.7500 to -0.5072, Q (df = 29) = 616.2937, $p < 0.0001$, I^2 = 95.53%). The results indicate that, under both the random-effects and fixed-effects models, the adult lifespan of *Spodoptera exigua* significantly shortens with increasing temperature. The black squares represent the cumulative effect size for each variable, and the black horizontal lines represent the 95% confidence intervals. In both Panel A and Panel B, the red line indicates the cumulative effect size.

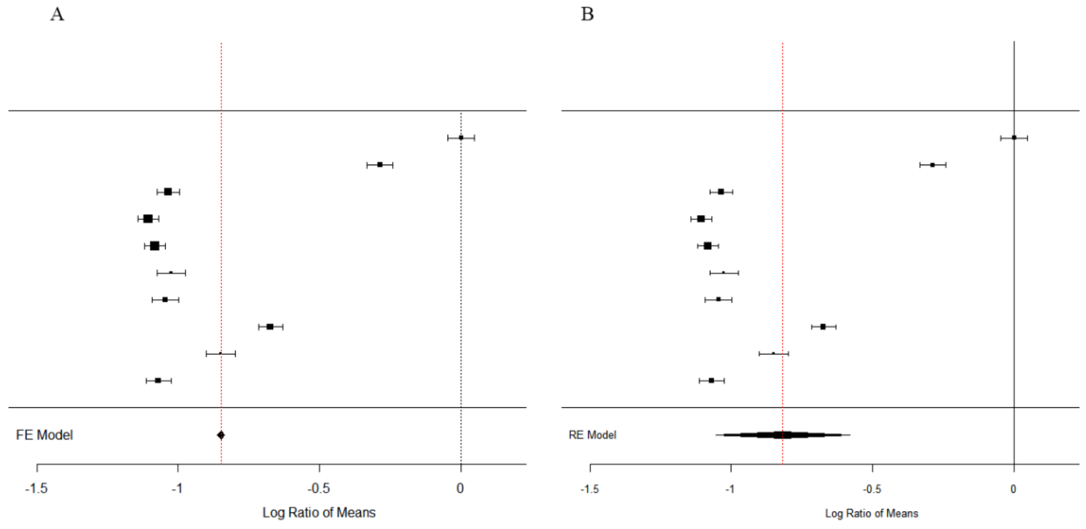

Figure S13 Comparison of effect sizes between the random-effects model and fixed-effects model (Panel A shows the results from the fixed-effects model, E = -0.8493, 95% confidence interval from -0.8493 to -0.8356, Q (df = 9) = 2513.7223, $p < 0.0001$, I^2 = 99.64%. Panel B shows the results from the random-effects model, E = -0.8175, 95% confidence interval from -1.0558 to -0.5793, Q (df = 9) = 2513.7223, $p < 0.0001$, I^2 = 99.67%). The results indicate that, under both the random-effects and fixed-effects models, the generation time of *Spodoptera exigua* significantly shortens with increasing temperature. The black squares represent the cumulative effect size for each variable, and the black horizontal lines represent the 95% confidence intervals. In both Panel A and Panel B, the red line indicates the cumulative effect size.
